# *Plasmodium vivax *lineages: geographical distribution, tandem repeat polymorphism, and phylogenetic relationship

**DOI:** 10.1186/1475-2875-10-374

**Published:** 2011-12-19

**Authors:** Surendra K Prajapati, Hema Joshi, Sneh Shalini, Manuel A Patarroyo, Rossarin Suwanarusk, Ashwani Kumar, Surya K Sharma, Alex Eapen, Vas Dev, Rajendra M Bhatt, Neena Valecha, Francois Nosten, Moshahid A Rizvi, Aditya P Dash

**Affiliations:** 1 Molecular Biology Division, National Institute of Malaria Research (NIMR), Sector-8, Dwarka, New Delhi, India; 2 Molecular Biology Department, Fundación Instituto de Inmunología de Colombia, Bogotá, Colombia; 3 School of Medicine and Health Sciences, Universidad del Rosario, Bogotá, Colombia; 4 Singapore Immunology Network (SIgN), A*STAR, Biopolis, Singapore; 5 NIMR field station, Goa, India; 6 NIMR field station, Rourkela, India; 7 NIMR field station, Chennai, India; 8 NIMR field station, Sonapur, India; 9 NIMR field station, Raipur, India; 10 Shoklo Malaria Research Unit, Mae Sot, Tak, Thailand; 11 Faculty of Tropical Medicine, Mahidol University, Bangkok, Thailand; 12 Centre for Tropical Medicine, Nuffield Department of Clinical Medicine, University of Oxford, CCVTM, Oxford, UK; 13 Department of Biosciences, Jamia Millia Islamia University, New Delhi, India; 14 World Health Organization, New Delhi, India

## Abstract

**Background:**

Multi-drug resistance and severe/complicated cases are the emerging phenotypes of vivax malaria, which may deteriorate current anti-malarial control measures. The emergence of these phenotypes could be associated with either of the two *Plasmodium vivax *lineages. The two lineages had been categorized as Old World and New World, based on geographical sub-division and genetic and phenotypical markers. This study revisited the lineage hypothesis of *P. vivax *by typing the distribution of lineages among global isolates and evaluated their genetic relatedness using a panel of new mini-satellite markers.

**Methods:**

*18S SSU rRNA S-type *gene was amplified from 420 *Plasmodium vivax *field isolates collected from different geographical regions of India, Thailand and Colombia as well as four strains each of *P. vivax *originating from Nicaragua, Panama, Thailand (Pak Chang), and Vietnam (ONG). A mini-satellite marker panel was then developed to understand the population genetic parameters and tested on a sample subset of both lineages.

**Results:**

*18S SSU rRNA S-type *gene typing revealed the distribution of both lineages (Old World and New World) in all geographical regions. However, distribution of *Plasmodium vivax *lineages was highly variable in every geographical region. The lack of geographical sub-division between lineages suggests that both lineages are globally distributed. Ten mini-satellites were scanned from the *P. vivax *genome sequence; these tandem repeats were located in eight of the chromosomes. Mini-satellites revealed substantial allelic diversity (7-21, *AE *= 14.6 ± 2.0) and heterozygosity (*He *= 0.697-0.924, *AE *= 0.857 ± 0.033) per locus. Mini-satellite comparison between the two lineages revealed high but similar pattern of genetic diversity, allele frequency, and high degree of allele sharing. A Neighbour-Joining phylogenetic tree derived from genetic distance data obtained from ten mini-satellites also placed both lineages together in every cluster.

**Conclusions:**

The global lineage distribution, lack of genetic distance, similar pattern of genetic diversity, and allele sharing strongly suggested that both lineages are a single species and thus new emerging phenotypes associated with vivax malaria could not be clearly classified as belonging to a particular lineage on basis of their geographical origin.

## Background

Malaria is a life-threatening parasitic disease which results in 247 million clinical episodes and nearly one million deaths annually [1]. *Plasmodium falciparum *is the most lethal human malaria species and causes malignant malaria globally, while *Plasmodium vivax *is the most prevalent species outside Africa, causing widespread morbidity and rarely severe and fatal [2-7]. India accounts for 77% of total malaria in Southeast Asia, *P. vivax *being responsible for more than 50% of malarial cases in India annually [8].

The population genetic structure of parasite species inhabiting widely separated geographical regions defines the level of population sub-division that is the basis for allopatric speciation. *Plasmodium falciparum *microsatellite studies have revealed strong bio-geographic population sub-structuring [9-11]. Likewise, *P. vivax *isolates have displayed weak population sub-structuring and shown a global population structure in several studies [12-14]. Li et al. categorized *P. vivax *into two distinct lineages for the first time as being Old World and New World [15] on the basis of linkage between phenotypic and genetic markers in a wide range of *P. vivax *strains. The distinction between the two lineages was (1) distinct geographic distribution, (2) differences in mosquito transmission potential, (3) fixation of the *S-type *polymorph from the *18S *small subunit ribosomal protein gene, and (4) nucleotide substitution in open reading frame 470 (*orf 470*) from the apicoplast genome. *Plasmodium vivax *isolates from the American continent were designated as New World and parasite isolates from rest of the world as Old World and they claimed that the New World lineage should be designated as a new subspecies of *P. vivax *[15].

Neutral genetic loci (tandem repeats) are considered potential genetic markers for unravelling an organism's genetic structure, population and evolutionary history. Many population studies have exploited polymorphism in two kinds of neutral loci: SNPs in putative housekeeping genes, and length polymorphism in microsatellites and mini-satellites. Genome sequence of *P. vivax *has revealed huge number of mini-satellites [16]. Genome-wide polymorphic microsatellite and mini-satellite markers have been widely used for detecting human malaria parasite population structure and genetic diversity [12,14,17-20].

The emergence of multi-drug resistance and vivax malaria-associated severe/complicated phenotypes now being reported from most vivax malaria dominated countries [2-7] could lead to the assumption that either Old World or New World parasites may have made a major contribution towards these phenotypes' emergence if both lineages (Old World and New World) are really genetically different. Furthermore, preliminary work carried out in the National Institute of Malaria Research (NIMR) in India has revealed the presence of both lineages on the Indian subcontinent [21], thus challenging Li et al's hypothesis. The established *P. vivax *lineage hypothesis has thus been questioned in this paper to uncover *P. vivax *lineages' genetic relatedness using a panel of neutral genomic markers.

## Methods

### Parasite isolates and DNA isolation

Four hundred and twenty *Plasmodium vivax *field isolates were analysed; they were collected from nine geographical populations from the Indian subcontinent (N = 354), three Colombian regions (N = 30), and a single Thai region (N = 36), as well as four parasite strains each from Nicaragua, Panama, Thailand (Pak Chang), and Vietnam (ONG). Details of individual study sites such as location, parasite and vector species prevalence and disease transmission pattern, are given in Additional file 1. Genomic DNA was extracted using QIAamp mini DNA kit (Qiagen, Germany) from microscopically diagnosed *P. vivax*-positive blood spotted on Whatman filter paper (3 mm) strips. Three punches (5 mm diameter) of dried blood spots were used for DNA isolation for each sample, as per kit manufacturer's instructions. DNA was eluted in sterile triple distilled water and stored at -20°C for future use.

### Ethics statement

The National Institute of Malaria Research's ethics committee approved the study protocol and all blood spots were collected with the written consent of the patients and/or and their legal guardians.

### Identification of mini-satellites

Tandem Repeat Finder (TRF) software version 4.00 [22] was used for the identification of mini-satellites from *P. vivax *genome sequence. Contig-wise sequence of *P. vivax *genome was scanned for up to 30 nucleotide repeat motif. TRF scan result was filtered according to several parameters such as alignment maximum score to individual repeat motif, minimum number of Indel (insertions/deletions), unit of repeat motif, number of repeat motif (copy number), less number of mismatch in repeats and high percent identity. All the parameters were given an equal weight during mini-satellite selection and 8-15 nucleotide repeat motifs with 95-100% identity were selected.

### PCR amplification and tandem repeat genotyping

A touchdown PCR method was used for the amplifying the *18S SSU rRNA S-type *gene from *P. vivax *isolates. Touchdown PCR conditions were: initial denaturation 95°C/5 min, and ten cycles consisting of: denaturation 95°C/20 s (s), annealing 65°C/30s and extension at 72°C/45 s. Annealing temperature was reduced by 1°C in each cycle until reaching 55°C. At this annealing temperature (55°C), 35 additional cycles were run, followed by a final extension step at 72°C/10 min. PCR conditions for mini-satellite amplification were as follows: initial denaturation 95°C/5 min, denaturation 95°C/30s, specific annealing temperature depending on the selected marker/30s and extension at 72°C/45 s for 35 cycles, and a final extension at 72°C/10 min. Annealing temperatures for each mini-satellite and PCR primer sequences of selected genes/loci are shown in Table 1. All PCR amplification reactions were carried out in a 20.0 μL final volume; 1-2 μL (~ 3-5 ng) template DNA, 10 pM each primer, 2X Master Mix (10.0 μL) (Promega or Qiagen).

**Table 1:** *Plasmodium vivax *primers list and their annealing temperature

Gene/locus	Primer name	Primer sequences (5'-3')	Annealing Tm (°C)
18S SSU rRNA	SSU-F	ATGAACGAGATCTTAACCTGC	65/55
	SSU-R	CATCACGATATGTA5TGATAAAGATTACC	
MiniSat-1	Mini1-F	ATGCTTCATTGGGTCCAC	50
	Mini1-R	TCGAACAGGACAATGCTG	
MiniSat-2	Mini2-F	TCACCGGTGGGTCCTTCG	50
	Mini2-R	GCAGCGACGAACCGTCAC	
MiniSat-5	Mini5-F	CAACCTGCAGAGCAATGC	55
	Mini5-R	ACGTTTCTGGGCGACTTC	
MiniSat-6	Mini6-F	TTGTGCTGTGCTGTGCTG	55
	Mini6-R	ACGGTTGGTATGGTCAGG	
MiniSat-8	Mini8-F	AGCCACAATCCCAACTGC	52
	Mini8-R	TGGTGGTTGTGACTCTAG	
MiniSat-11	Mini11-F	GGCACAGTGATCATATTCG	55
	Mini11-R	GCGGGTACATAACGCATG	
MiniSat-13	Mini13-F	GGCACATGAACTTTTCGG	52
	Mini13-R	TTCACCATGGTCCCTTCG	
MiniSat-14	Mini14-F	CTCTTCGTCGCGTCCAGG	52
	Mini14-R	CAGGGTATCCACGACCAG	
MiniSat-16	Mini16-F	TATGTACTACCTCCACCC	52
	Mini16-R	AGCGCGAATATGCATACG	
PvCDPK	CDPK-F	CGCCTCTTTTTCGAGCCC	55
	CDPK-R	CTGCGCCTTCCGCGTCTT	

High-resolution metaphor agarose (3-4% gel) was used for mini-satellite allele sizing. A fixed electrophoresis protocol (2 h @ 80 V) was used for all experiments for consistency in allele sizing. A 20 bp DNA ladder and Genetool software were used (SynGene Inc., UK) for allele sizing.

### Single clone infection typing

As multi-clone isolates could mislead correct genotyping and lead to over-estimating genetic diversity in a multi-locus genotyping study, *Pvmsp-3α *PCR-RFLP analysis was used to identify single- and multi-clone infections [23]; only single-clone infection samples (n = 96) originating from five geographical regions of India (Delhi, Chennai, Nadiad, Panna and Sonapur) were used for multi-locus genotyping.

### Measures of genetic diversity and phylogenetic analysis

Mini-satellite genetic diversity was measured by calculating virtual heterozygosity (*He*) for each locus using microsatellite analyzer (MSA) version 4.00 [24]. Virtual heterozygosity (*He*) was defined as [n/(n - 1)] [1 - Σp_i_^2^], where n was the number of isolates analysed and pi was the frequency of the i^th ^allele in the population. MEGA version 4.00 [25,26] was used for un-rooted Neighbour-Joining phylogenetic tree reconstruction method to understand the genetic relatedness of *P. vivax S type-1 *and *S type-2 *lineages.

## Results

### Geographic mapping of *S type-1 *and *S type-2 *lineages

**Figure 1:**
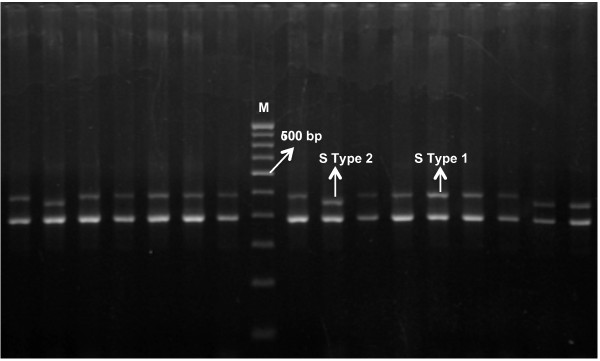
**Gel image of *18S SSU rRNA S-type *gene polymorphism distinguishing *S type-1 *(Old World lineage) and *S type-2 *(New World lineage)**.

**Figure 2:**
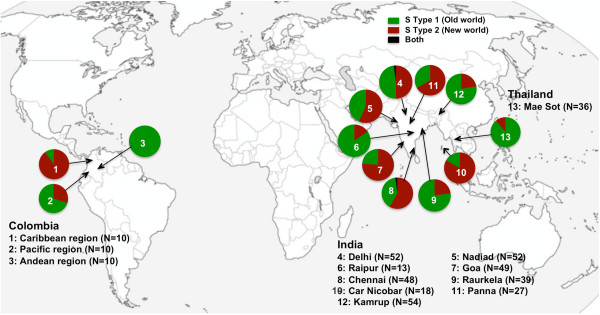
***S type-1 *and *S type-2 *lineages distribution among global *Plasmodium vivax *isolates**.

The *18S SSU rRNA S-type *gene was amplified from 420 *P. vivax *isolates from the Indian subcontinent (N = 354), Thailand (N = 36) and Colombia (N = 30) and four strains each from Nicaragua, Panama, Thailand (Pak Chang), and Vietnam (ONG). Amplified *S type 18S SSU rRNA *DNA consisted of two PCR fragments in each sample, one having consistent size called *A-type *(390 bp) and a second variable sized fragment called *S-type *(either 480 bp or 450 bp). 480 bp and 450 bp PCR fragments were designated as *S type-1 *and *S type-2*, respectively (Figure 1) and served as molecular markers for identifying Old World and New World lineages of *P. vivax*, respectively [15]. A fair number of both *P. vivax *lineages were found on the Indian subcontinent, Thailand and Colombia; however, their distribution in geographical regions was highly variable (Figure 2). By contrast, two *P. vivax *strains from Central America (Nicaragua and Panama) were genotyped as being *S type-2 *lineage and two strains from Southeast Asia (Thailand and Vietnam) as *S type-1 *lineage, indicating geographical sub-division observed between Old World and New World *P. vivax *strains could be by chance. The *P. vivax *lineage distribution pattern for global isolates suggested a lack of parasite bio-geographic structure between New World and Old World regions; varied *P. vivax *lineage distribution throughout geographical regions was not associated with vivax or falciparum malaria or vector species prevalence (Table 2). High Old World lineage frequency was found with the *Anopheles minimus *malaria vector in the north-eastern part of India and Thailand; however, this pattern was not consistent since one study site from Colombia revealed 100% Old World-type prevalence with different malaria vectors (*Anopheles albimanus *and *Anopheles darlingi*).

**Table 2:** Regional distribution of *S type-1 *and *S type-2 *lineages and malaria vector prevalence

Regions	Major malaria parasites and vectors	Sample (n)	*S type-2 *(%)	*S type-1 *(%)	Both (%)
**India**					
Delhi	Pf & Pv, *An. stephensi, An. culicifacies*	52	50	48.08	1.92
Nadiad	Pf & Pv, *An. culicifacies, An. stephensi*	52	55.76	42.3	1.92
Panna	Pf & Pv, *An. culicifacies, An. fluvitalis*	27	66.66	33.34	-
Raipur	Pf, *An. culicifacies, An. fluvitalis*	13	15.38	84.62	-
Rourkela	Pf, *An. fluvitalis, An. culicifacies*	39	23.07	76.93	-
Goa	Pf & Pv, *An. stephensi*	51	76.47	23.52	-
Chennai	Pv, *An. stephensi*	48	58.33	39.58	2.08
Kamrup	Pf & Pv, *An. minimus, An. dirus*	54	22.23	77.77	-
Car Nicobar	Pf & Pv, *An. sandaicus*	18	83.33	16.67	-
Thailand					
Mae Sot	Pf & Pv, *An. dirus*	36	11.11	88.89	-
Colombia					
Andean region	Pf & Pv, *An. albimanus, An. darlingi*	10	0	100	-
Pacific region	Pf & Pv, *An. albimanus, An. neivai*	10	90	10	-
Caribbean region	Pf & Pv, *An. albimanus, An. nuñeztovari*	10	30	70	-
**Total**		420	46.19	53.09	0.72

Both lineages' global distribution contradicted Li *et al's *Old and New World lineages hypothesis; however, the sympatric distribution of lineages was not sufficient to refute the two-lineage hypothesis; rather, understanding their genetic relatedness would seem to be more important. Ninety-six single-clone isolates (48 *S type-1 *and 48 *S type-2*) were selected and characterized using a panel of mini-satellite markers to unravel genetic relatedness between *P. vivax *lineages.

### Pattern of genetic diversity between *S type-1 *and *S type-2 *lineages

**Figure 3:**
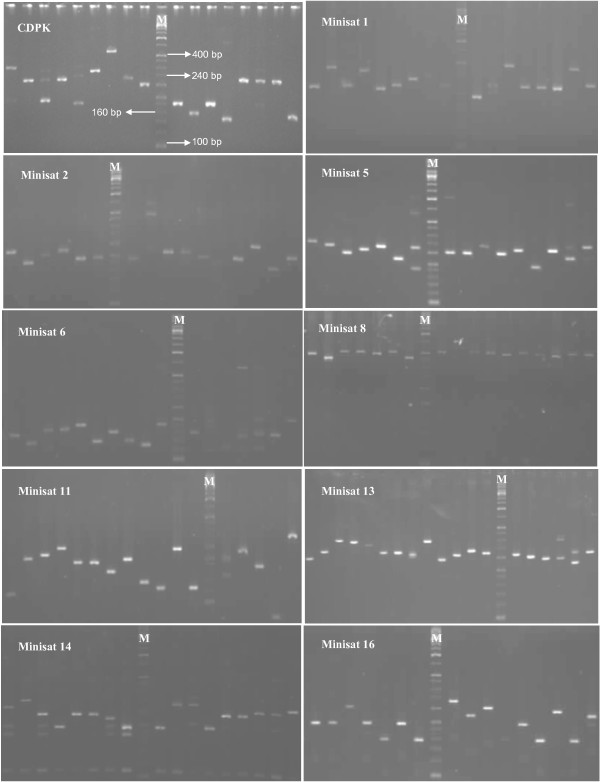
**Gel images of individual mini-satellite polymorphism among field isolates of *Plasmodium vivax***.

Ten mini-satellites were scanned from the *P. vivax *genome sequence; these tandem repeats were located in eight of the chromosomes (Table 3). All selected mini-satellites were highly polymorphic (Figure 3). A substantial number of alleles per locus (7-21, *AE *= 14.6 ± 2.0) were observed at all mini-satellites under study except for a single locus (Minisat 8), which showed seven alleles. However, average heterozygosity was very high at all studied loci in *P. vivax *field isolates (*He *= 0.697-0.924, *AE *= 0.857 ± 0.033).

**Table 3:** Characteristic features of *Plasmodium vivax *mini and microsatellite markers

Locus name	**Chro No**.	Repeat size	Repeat Unit	**Copy No**.	Size (bp)
PvCDPK	4	12	ATTTTGCTTTCC	25	375
MiniSat 1	2	15	TTTTTCCCCATCTCA	12.3	279
MiniSat 2	2	11	AACAAAAAAAA	15.6	255
MiniSat 5	2	15	GGGGAGAGCGGCAAA	10.1	254
MiniSat 6	10	11	TTTTCTTCCTT	11.7	172
MiniSat 8	10	12	AACCAGAAATGG	30.2	404
MiniSat 11	14	12	TGCTGCTCCGAC	12.8	197
MiniSat 13	14	9	AGGTTAAGC	11.8	258
MiniSat 14	6	12	GTTGCCGCCGTG	16	287
MiniSat 16	8	12	ATGTACCTACTG	16.2	262

**Figure 4:**
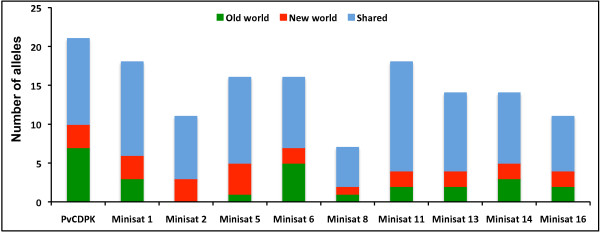
**Allele sharing in mini-satellites between *S type-1 *and *S type-2 *lineages**.

**Figure 5:**
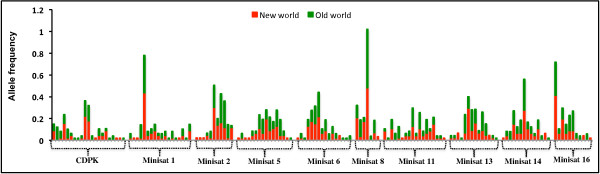
**Allele frequency sharing at mini-satellite marker between two lineages**.

**Figure 6:**
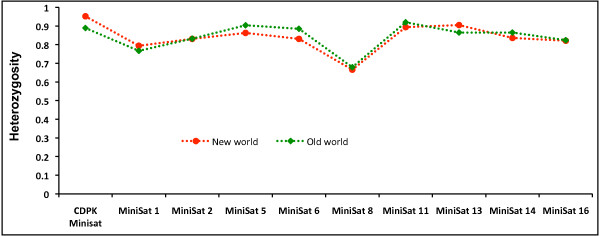
**Degree of heterozygosity per locus in two lineages of *Plasmodium vivax***.

Allelic diversity, frequency distribution and sharing as well as heterozygosity were compared between *P. vivax *lineages. Total allelic diversity of mini-satellites was high and similar in both lineages. A high degree of alleles in mini-satellites (66%, 96/146) was shared between both lineages (Figure 4). Allele frequency per locus varied considerably; however, allele frequencies were evenly distributed between the lineages (Figure 5). Heterozygosity analysis revealed a similar mini-satellite genetic diversity pattern between both lineages (Figure 6). Thus, allelic diversity, sharing, distribution frequency and heterozygosity between both lineages revealed a similar pattern; however, a high degree of genetic diversity could only be expected from older species compared to newly evolved/established species.

### Genetic relationship of *S type-1 *and *S type-2 *lineages of *P. vivax*

**Figure 7:**
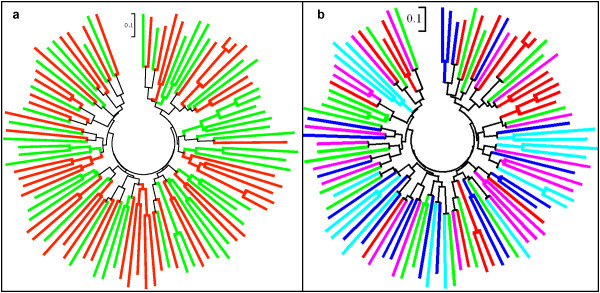
**N-J phylogenetic tree constructed using *S type-1 *and *S type-2 *lineage multi-locus microsatellite genetic distance**. A: phylogenetic relationship between two lineages and, green and red lines indicate *S type-1 *and *S type-2 *lineages, b: genetic structure of *Plasmodium vivax *isolates among five geographical regions (five colors).

Unrooted Neighbour-Joining phylogenetic trees were constructed using 10 mini-satellite multi-locus genetic distance data. The phylogenetic tree showed five clusters having a high degree of divergence among isolates; however, no cluster was specific for any of the two lineages (Figure 7), indicating no distinction between both lineages. As per Li *et al*'s hypothesis, it was assumed that *S type-1 *and *S type-2 *isolates would have their separate phylogenetic cluster.

## Discussion

This study reports large-scale geographical mapping of the two *Plasmodium vivax *lineages on global isolates (Indian subcontinent, Thailand, and Colombia) and their genetic relatedness using a panel of neutral genomic markers. This study revealed that both *P. vivax *lineages have a global distribution and that their genetic structure has not been maintained between Old World and New World regions, and strongly suggests that both lineages are a population from a single species.

The global distribution of *P. vivax *Old World and New World lineages clearly demonstrates a lack of geographical subdivision; however, it cannot be concluded that they form a single species based only on a lack of geographical subdivision, as both lineages may have attained geographical sympatricity due to a gene flow mechanism. Their genetic relatedness was characterized by using 48 *S type-1 *and 48 *S type-2 *lineage samples to rule this out. The genetic relatedness analysis showed that both proposed genetic lineages were from the same *P. vivax *population based on the lack of genetic distance, similar genetic diversity magnitude, and no separate phylogenetic clade/cluster. As per Li et al., it was assumed that *S type-2 *lineage (New World) is a recently evolved species and it was expected that *S type-2 *lineage would be less diverse, and form a separate phylogenetic clade, if the hypothesis was correct. However, the panel of genomic markers consistently revealed similar patterns with all parameters used, providing no support for the hypothesis that *S type-2 *lineage is a subspecies of *P. vivax*. This clearly suggested that *S type 18S SSU rRNA *gene polymorphism (*S type-1 *and *S type-2*) was not representative of two *P. vivax *lineages/sub species, rather, that both are allelic variants. The lack of significant genetic distance and population structure between *P. vivax *isolates from Old world and New World regions, in several studies, are also in strong support of the findings here reported [12-14].

Questioning of the hypothesis of Li et al. is based on contrasting findings (i.e. a lack of geographical subdivision among global field isolates but not in parasite strains). The observations reported by Li et al. might have been due to their small sample size (17 isolates from 11 different countries) and their analysis being based only on parasite strains instead of field isolates. The regional prevalence of *S type-1 *and *S type-2 *could have been due to local vector and parasite adaptation. A recent study in Mexico by Joy et al. [19] uncovered the importance of local parasite and vector adaptation in genetic structuring of parasite populations. The effect of the mosquito's differential transmission potential, observed by Li et al., could have been the effect of local parasite-mosquito adaptation rather than speciation.

This is the first in-depth study dealing with *P. vivax *genetic diversity on the Indian subcontinent using mini-satellite marker, suggesting huge neutral genetic variation in Indian field isolates. The high degree of genetic polymorphism observed in tandem repeats agrees with earlier studies, which revealed tremendous genetic polymorphism among global *P. vivax *isolates [12,14,17,18,20,27]. Although the present study has revealed a higher level of genetic diversity compared to that previously reported for *P. vivax *mini-satellites [28], this might be due to the random mating nature of Indian *P. vivax *field isolates [29]. The higher degree of genetic polymorphism displayed by Indian *P. vivax *field isolates in earlier studies [21,29-34] also supports our findings. This study successfully uncovered a substantial amount of neutral genetic variation in field isolates, implying their potential use as molecular markers in identifying population structures and relapse and recrudescence infection studies.

## Conclusions

The findings have thus revealed that the *Plasmodium vivax *two-lineage hypothesis is unlikely; the dimorphism observed in *S type 18S SSU rRNA *gene represents only two alleles and both lineages are a single *P. vivax *species. The mini-satellite marker panel developed here is highly polymorphic and could be employed in therapeutic efficacy, infection relapse, and population structure studies.

## Competing interests

The authors declare that they have no competing interests.

## Authors' contributions

SKP: Experiment design, experimental work, data analysis, manuscript writing. SS, RS, MAP: Experimental work, data analysis, manuscript writing. AK, SKS, AE, VD, RMB, NV, FN: Sample collection, parasite identification, and experimental design. HJ: Conceptual design and supervision of work, manuscript writing. MMAR and APD: Overall supervision of work, and manuscript writing. All authors read and approved the final manuscript.

## Supplementary Material

Additional file 1 **Additional information**. Click here for file
