# Supplementary material for: Plasmodium vivax lineages: geographical distribution, tandem repeat polymorphism, and phylogenetic relationship
Source: Malar J. 2011 Dec 19;10:374. doi: 10.1186/1475-2875-10-374 (PMC3258263; doi:10.1186/1475-2875-10-374)
Supplement: Additional file 1 — Additional information. [file 1475-2875-10-374-S1.DOC]

**Additional information:**

**Study site details:**

**Indian subcontinent:**

**Delhi** (New Delhi), the capital of India located in the north, is subject to frequent influx of people from all over the country seeking jobs. Malaria in Delhi is transmitted by mosquito vectors *Anopheles stephensi* and *An. culicifacies* in urban and rural populations, respectively. *Plasmodium vivax* is the predominant malaria parasite species. *P. vivax* transmission occurs in two clear peaks, a lesser peak in March/April and a major peak in the post-monsoon months of July to October. *P. falciparum* transmission occurs during September to October until November and is interrupted during the winter months (December to January). *P. vivax* malaria in the Delhi region is characterized by a high relapse rate [1, 2].

**Nadiad** is a city in a malaria-endemic region in Gujarat state, located in western India. State malaria records reveal that malaria incidence was historically highest in the Kheda district (including Nadiad) in the form of epidemics. Both species of *Plasmodium* (*P. falciparum* and *P. vivax*) are present, though *P. vivax* is predominant (70-80%) and their transmission is mainly during post-monsoon months. Malaria in Nadiad is transmitted by the vectors *An. stephensi* and *An. culicifacies* in urban and rural populations, respectively. Relapses due to vivax malaria have been reported in Nadiad [3, 4].

**Panna** is an isolated, hilly, forested area in Madhya Pradesh state (Central India) and is dominated by tribal populations (mainly Gond). Malaria transmission occurs throughout the year with predominance of *P. falciparum*. *P. vivax* transmission is mainly during March to July. In recent years, the forested area has experienced several malarial epidemics [5-9]. *An. culicifacies* and *An. fluvitalis* are the major malaria vectors in this region.

**Raipur** (Chhattishgarh) is the second most malarial state after Orissa and is located in the central region of India. The area is dominated by tribal populations *e.g.,* Gond, Kanwar, Brinjhwar, Bhaina, Bhatra, Oraon. Frequent outbreaks of malaria in Raipur are recorded [5, 10, 11]. In Raipur, malaria is mainly transmitted by *An. culicifacies* although in forested areas *An. fluvitalis* is the main vector. The percentage of *P. falciparum* is significantly higher than *P. vivax*.

**Sundergarh (Orissa)** is located in eastern India. It is dominated by tribal populations *e.g.,* Munda, Oraon, Kharia, Kissan Bhuiyan. The area is hyper-endemic for malaria, with more than 85% of total malaria cases being *P. falciparum*. *P. vivax* accounts for about 10-15% and *P. malariae* for about 1%. It is a hilly, forested area, and malaria transmission is via the highly anthropophagic vector *An. fluvitalis.* Malaria transmission in this region is stable, especially in forested regions where chloroquine-resistant *P. falciparum* strains are highly prevalent [12].

**Goa** is a coastal area of tourist interest and is located in the southwest of the country. Goa is host to many migrant laborers who work on Goa’s extensive construction projects. To and fro movement of laborers between Goa and their homes introduces parasite strains from different areas into Goa. Malaria in Goa is dominated by *P. vivax;* however, cases of *P. falciparum* are also reported. In Goa, malaria is primarily transmitted along the coast by *An. stephensi.*

**Chennai** (Tamil Nadu) is a coastal metropolitan city located in the southern region of the country. Chennai is rife with urban malaria. Large numbers of overhead water tanks in the city provides good conditions for mosquito breeding. Malaria there is mainly (>90%) due to *P. vivax* and is transmitted by *An. stephensi*. Malaria transmission is stable in Chennai and occurs throughout the year. Recently, cases of chloroquine-resistant *P. falciparum* malaria have been reported in Tamil Nadu state [13].

**Car Nicobar** (Andaman & Nicobar Islands) is an island located in the Bay of Bengal. Andaman and Nicobar islands are the home of six tribes, namely Great Andamanese, Onges, Jarwas, Sentinelese, Nicobarese and Shompens [14]. The area is endemic for malaria and both *P. vivax* and *P. falciparum* are prevalent throughout the year. *An.* *sundaicus* is the malarial vector. Chloroquine-resistant strains of *P. falciparum* are common (National Vector Borne Disease Control Programme, [http://www.nvbdcp.gov.in](http://www.nvbdcp.gov.in/)).

**Kamrup** is in Assam state, located in the northeastern region of the country. *P. falciparum* is the predominant human malaria parasite, though rare cases of *P. vivax* are reported in epidemic form. Northeastern states of India are known to have high prevalence of multi-drug resistant strains [15] and the region is surrounded by international borders. The borders are very porous; illegal migration of people across the borders is common, facilitating import of drug-resistant strains. Malaria transmission vectors in this region are the highly anthropophagic mosquitoes *An. minimus* and *An. dirus.*

**Thailand:**

**Mae Sot** is in Tak province of western Thailand and shares a border with Myanmar to the west. Mae Sot is home to refugees from Myanmar and there is frequent influx of people seeking work. This region is highly endemic for malaria; chloroquine-resistant *P. falciparum* strains are prevalent. Both *P. falciparum* and *P. vivax* species are common; however, prevalence of the latter is slightly higher (> 50 %). Malaria in Mae Sot is mainly transmitted by *An. dirus*, a highly anthropophagic vector.

**Colombia:**

**Andean region:** This region covers the part of Colombia stretching from the southwest (bordering Ecuador) to its northeastern border with Venezuela, where *P. vivax* cases prevail, even though some *P. falciparum* malaria cases are reported per year [16]. The samples used in this study were collected from the Antioquia and Cundinamarca departments as they have very different endemicity rates (26,806 and 40 cases in 2009, respectively) [17]. The predominant vectors in this region, *An. albimanus* and *An. darlingi* [18-20], have been shown to be naturally infected by *P. vivax* [19].

**Caribbean region:** This is the most north-western continental and maritime area of Colombia. The most predominant parasite species is *P. vivax*, the same as in the Andean region. All samples were collected from the area around Puerto Libertador in the Córdoba department. This department presented 10,272 cases of *P. vivax* malaria and 2,966 cases caused by *P. falciparum* during 2009 [17]. *An. albimanus* was the most predominant vector in the area during 2005 [21] while *An. nuñeztovari* was the predominant species in this department during 2007, most of these mosquitoes having been naturally infected by *P. vivax* [19].

**Pacific region:** This area is located in western Colombia, bordering the Pacific Ocean. The samples were collected from near the towns of Istmina in Chocó and Tumaco in Nariño. Cases of *P. vivax* malaria are more frequent in the former while most cases of malaria in Nariño are attributed to *P. falciparum* [16]. 5,529 cases of *P. vivax* and 2,958 cases of *P. falciparum* malaria were reported for Choco in 2009 while 1,070 cases of *P. vivax* and 2,958 cases of *P. falciparum* malaria were reported for Nariño [17]. The *An. albimanus*, *An. neivai*, *An. nuñeztovari*, *An. albitarsis* and *An. apicimacula* species are the vectors found in this region [18, 20-22], *An. albimanus* and *An. neivai* being the main ones. Both *An. albimanus* and *An. neivai* have been shown to be naturally infected by *P. vivax* [21, 23].

**References**
